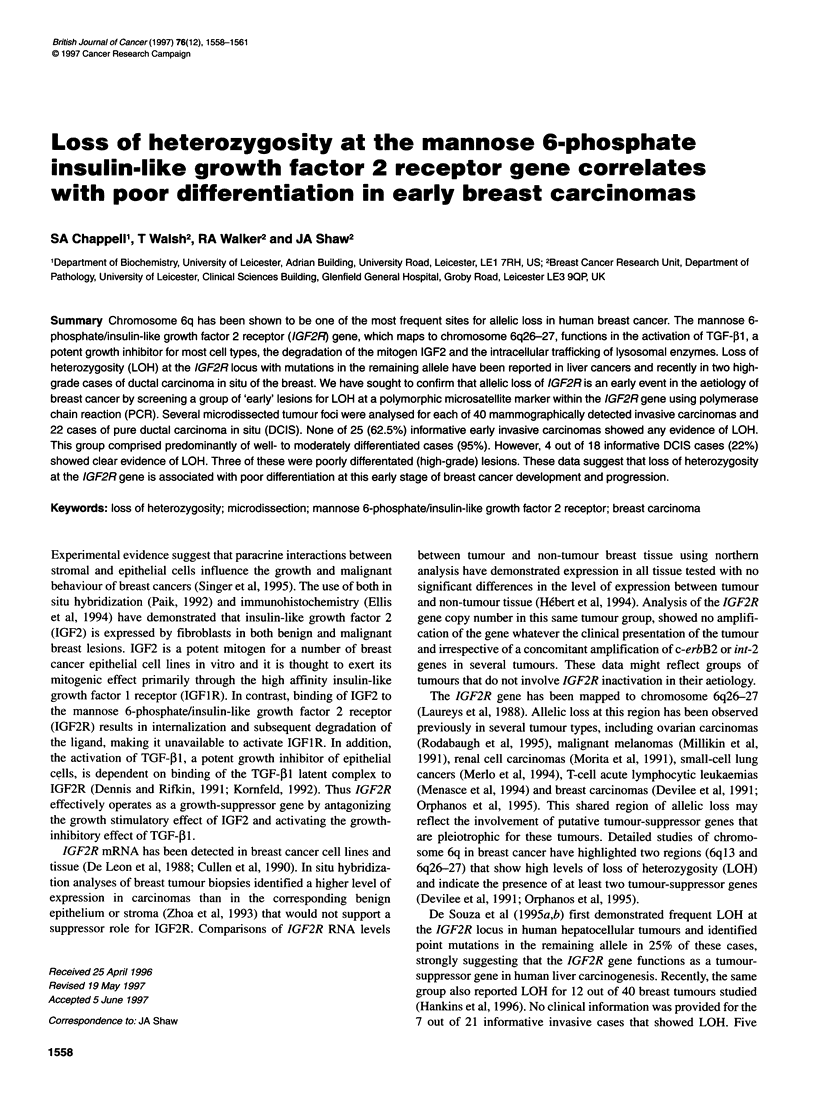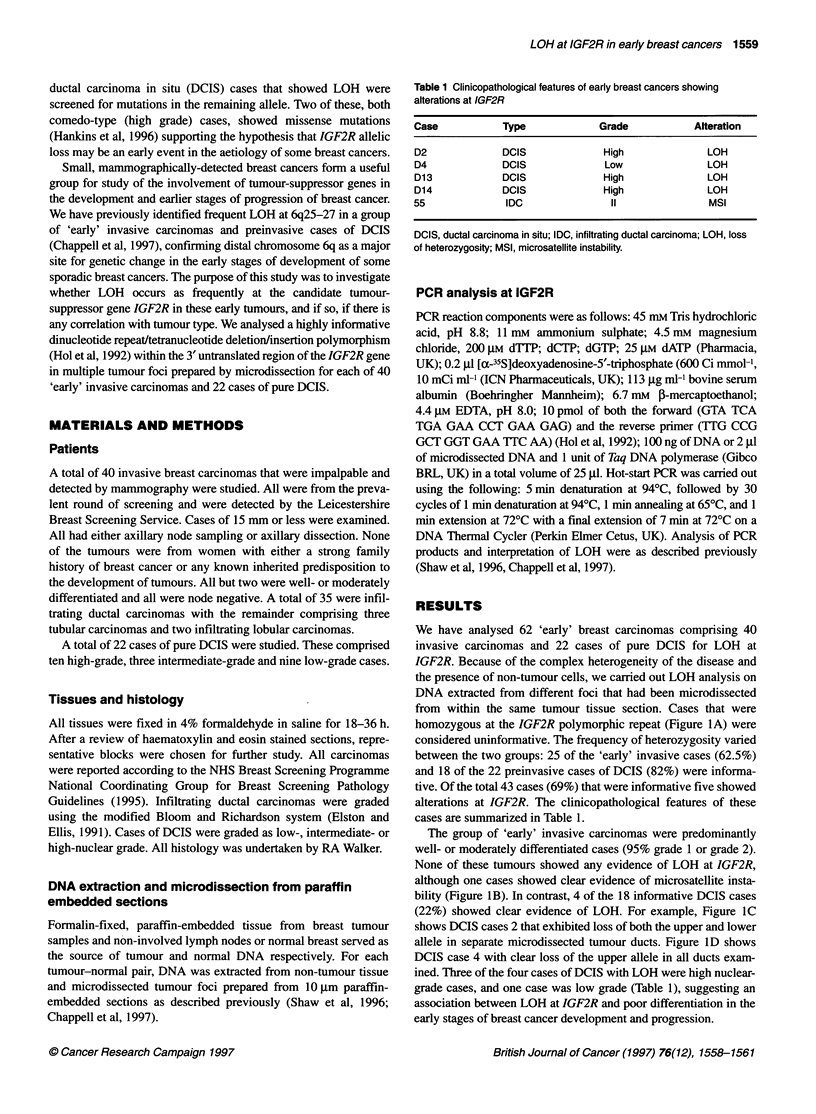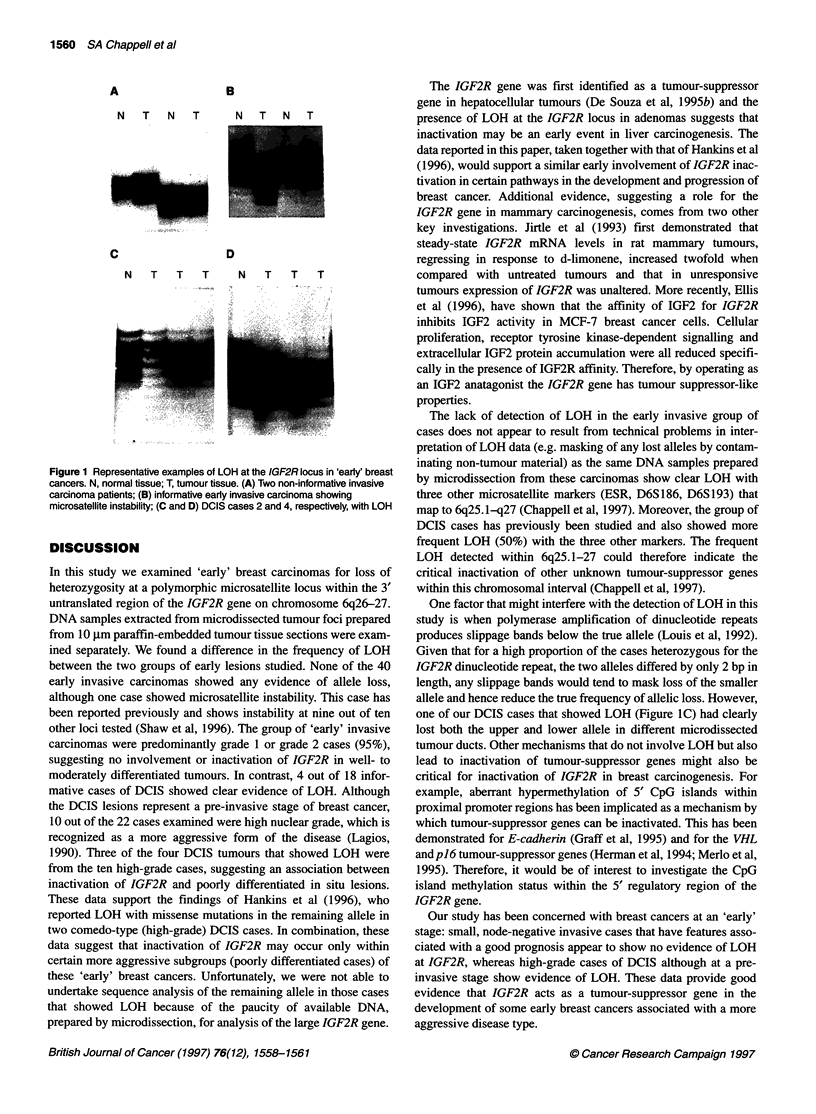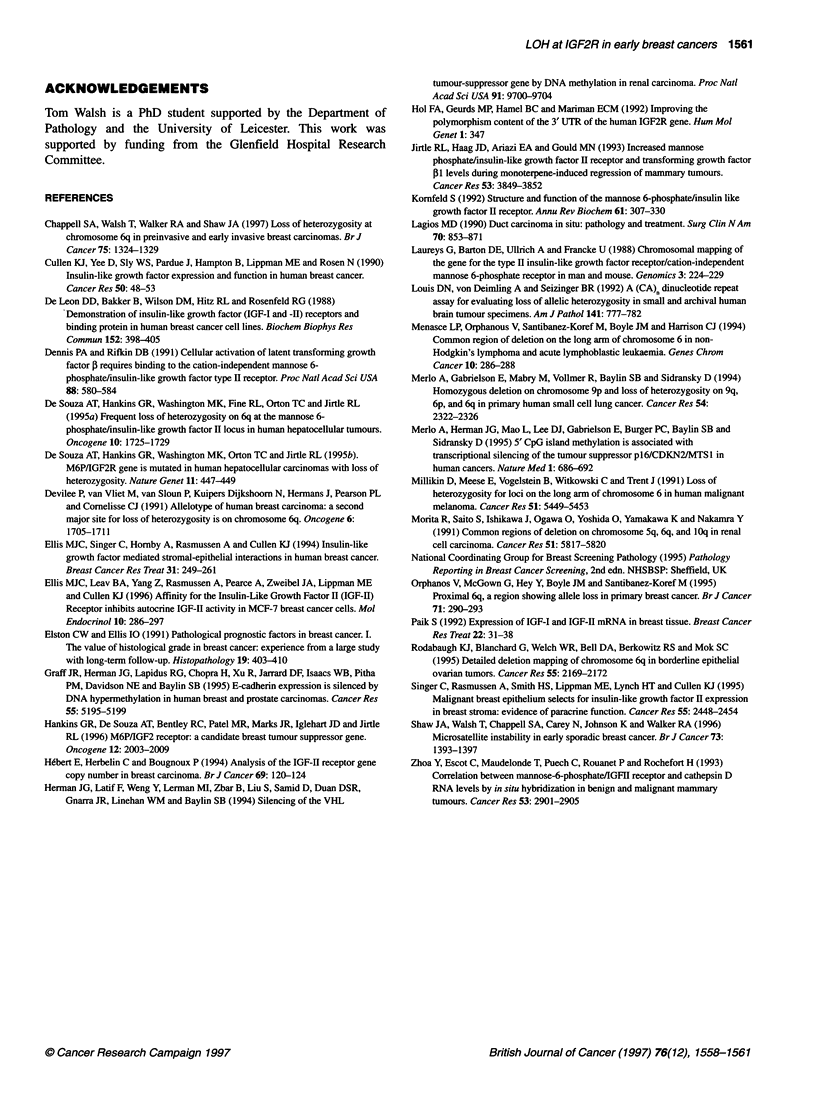# Loss of heterozygosity at the mannose 6-phosphate insulin-like growth factor 2 receptor gene correlates with poor differentiation in early breast carcinomas.

**DOI:** 10.1038/bjc.1997.596

**Published:** 1997

**Authors:** S. A. Chappell, T. Walsh, R. A. Walker, J. A. Shaw

**Affiliations:** Department of Biochemistry, University of Leicester, UK.

## Abstract

**Images:**


British Journal of Cancer (1997) 76(12), 1558–1561
© 1997 Cancer Research Campaign

### Loss of heterozygosity at the mannose 6-phosphate insulin-like growth factor 2 receptor gene correlates with poor differentiation in early breast carcinomas

**SA Chappell[1], T Walsh[2], RA Walker[2] and JA Shaw[2]**

[1]Department of Biochemistry, University of Leicester, Adrian Building, University Road, Leicester, LE1 7RH, US; [2]Breast Cancer Research Unit, Department of Pathology, University of Leicester, Clinical Sciences Building, Glenfield General Hospital, Groby Road, Leicester LE3 9QP, UK

**Summary** Chromosome 6q has been shown to be one of the most frequent sites for allelic loss in human breast cancer. The mannose 6-phosphate/insulin-like growth factor 2 receptor (*IGF2R*) gene, which maps to chromosome 6q26–27, functions in the activation of TGF-β1, a potent growth inhibitor for most cell types, the degradation of the mitogen IGF2 and the intracellular trafficking of lysosomal enzymes. Loss of heterozygosity (LOH) at the *IGF2R* locus with mutations in the remaining allele have been reported in liver cancers and recently in two high-grade cases of ductal carcinoma in situ of the breast. We have sought to confirm that allelic loss of *IGF2R* is an early event in the aetiology of breast cancer by screening a group of 'early' lesions for LOH at a polymorphic microsatellite marker within the *IGF2R* gene using polymerase chain reaction (PCR). Several microdissected tumour foci were analysed for each of 40 mammographically detected invasive carcinomas and 22 cases of pure ductal carcinoma in situ (DCIS). None of 25 (62.5%) informative early invasive carcinomas showed any evidence of LOH. This group comprised predominantly of well- to moderately differentiated cases (95%). However, 4 out of 18 informative DCIS cases (22%) showed clear evidence of LOH. Three of these were poorly differentated (high-grade) lesions. These data suggest that loss of heterozygosity at the *IGF2R* gene is associated with poor differentiation at this early stage of breast cancer development and progression.

**Keywords:** loss of heterozygosity; microdissection; mannose 6-phosphate/insulin-like growth factor 2 receptor; breast carcinoma

Experimental evidence suggest that paracrine interactions between stromal and epithelial cells influence the growth and malignant behaviour of breast cancers (Singer et al, 1995). The use of both in situ hybridization (Paik, 1992) and immunohistochemistry (Ellis et al, 1994) have demonstrated that insulin-like growth factor 2 (IGF2) is expressed by fibroblasts in both benign and malignant breast lesions. IGF2 is a potent mitogen for a number of breast cancer epithelial cell lines in vitro and it is thought to exert its mitogenic effect primarily through the high affinity insulin-like growth factor 1 receptor (IGF1R). In contrast, binding of IGF2 to the mannose 6-phosphate/insulin-like growth factor 2 receptor (IGF2R) results in internalization and subsequent degradation of the ligand, making it unavailable to activate IGF1R. In addition, the activation of TGF-β1, a potent growth inhibitor of epithelial cells, is dependent on binding of the TGF-β1 latent complex to IGF2R (Dennis and Rifkin, 1991; Kornfeld, 1992). Thus *IGF2R* effectively operates as a growth-suppressor gene by antagonizing the growth stimulatory effect of IGF2 and activating the growth-inhibitory effect of TGF-β1.

*IGF2R* mRNA has been detected in breast cancer cell lines and tissue (De Leon et al, 1988; Cullen et al, 1990). In situ hybridization analyses of breast tumour biopsies identified a higher level of expression in carcinomas than in the corresponding benign epithelium or stroma (Zhoa et al, 1993) that would not support a suppressor role for IGF2R. Comparisons of *IGF2R* RNA levels between tumour and non-tumour breast tissue using northern analysis have demonstrated expression in all tissue tested with no significant differences in the level of expression between tumour and non-tumour tissue (Hébert et al, 1994). Analysis of the *IGF2R* gene copy number in this same tumour group, showed no amplification of the gene whatever the clinical presentation of the tumour and irrespective of a concomitant amplification of c-*erb*B2 or *int*-2 genes in several tumours. These data might reflect groups of tumours that do not involve *IGF2R* inactivation in their aetiology.

The *IGF2R* gene has been mapped to chromosome 6q26–27 (Laureys et al, 1988). Allelic loss at this region has been observed previously in several tumour types, including ovarian carcinomas (Rodabaugh et al, 1995), malignant melanomas (Millikin et al, 1991), renal cell carcinomas (Morita et al, 1991), small-cell lung cancers (Merlo et al, 1994), T-cell acute lymphocytic leukaemias (Menasce et al, 1994) and breast carcinomas (Devilee et al, 1991; Orphanos et al, 1995). This shared region of allelic loss may reflect the involvement of putative tumour-suppressor genes that are pleiotrophic for these tumours. Detailed studies of chromosome 6q in breast cancer have highlighted two regions (6q13 and 6q26–27) that show high levels of loss of heterozygosity (LOH) and indicate the presence of at least two tumour-suppressor genes (Devilee et al, 1991; Orphanos et al, 1995).

De Souza et al (1995*a*,*b*) first demonstrated frequent LOH at the *IGF2R* locus in human hepatocellular tumours and identified point mutations in the remaining allele in 25% of these cases, strongly suggesting that the *IGF2R* gene functions as a tumour-suppressor gene in human liver carcinogenesis. Recently, the same group also reported LOH for 12 out of 40 breast tumours studied (Hankins et al, 1996). No clinical information was provided for the 7 out of 21 informative invasive cases that showed LOH. Five

*Received 25 April 1996*
*Revised 19 May 1997*
*Accepted 5 June 1997*

*Correspondence to:* JA Shaw ductal carcinoma in situ (DCIS) cases that showed LOH were screened for mutations in the remaining allele. Two of these, both comedo-type (high grade) cases, showed missense mutations (Hankins et al, 1996) supporting the hypothesis that *IGF2R* allelic loss may be an early event in the aetiology of some breast cancers.

Small, mammographically-detected breast cancers form a useful group for study of the involvement of tumour-suppressor genes in the development and earlier stages of progression of breast cancer. We have previously identified frequent LOH at 6q25–27 in a group of 'early' invasive carcinomas and preinvasive cases of DCIS (Chappell et al, 1997), confirming distal chromosome 6q as a major site for genetic change in the early stages of development of some sporadic breast cancers. The purpose of this study was to investigate whether LOH occurs as frequently at the candidate tumour-suppressor gene *IGF2R* in these early tumours, and if so, if there is any correlation with tumour type. We analysed a highly informative dinucleotide repeat/tetranucleotide deletion/insertion polymorphism (Hol et al, 1992) within the 3′ untranslated region of the *IGF2R* gene in multiple tumour foci prepared by microdissection for each of 40 'early' invasive carcinomas and 22 cases of pure DCIS.

### MATERIALS AND METHODS

### Patients

A total of 40 invasive breast carcinomas that were impalpable and detected by mammography were studied. All were from the prevalent round of screening and were detected by the Leicestershire Breast Screening Service. Cases of 15 mm or less were examined. All had either axillary node sampling or axillary dissection. None of the tumours were from women with either a strong family history of breast cancer or any known inherited predisposition to the development of tumours. All but two were well- or moderately differentiated and all were node negative. A total of 35 were infiltrating ductal carcinomas with the remainder comprising three tubular carcinomas and two infiltrating lobular carcinomas.

A total of 22 cases of pure DCIS were studied. These comprised ten high-grade, three intermediate-grade and nine low-grade cases.

### Tissues and histology

All tissues were fixed in 4% formaldehyde in saline for 18–36 h. After a review of haematoxylin and eosin stained sections, representative blocks were chosen for further study. All carcinomas were reported according to the NHS Breast Screening Programme National Coordinating Group for Breast Screening Pathology Guidelines (1995). Infiltrating ductal carcinomas were graded using the modified Bloom and Richardson system (Elston and Ellis, 1991). Cases of DCIS were graded as low-, intermediate- or high-nuclear grade. All histology was undertaken by RA Walker.

### DNA extraction and microdissection from paraffin embedded sections

Formalin-fixed, paraffin-embedded tissue from breast tumour samples and non-involved lymph nodes or normal breast served as the source of tumour and normal DNA respectively. For each tumour–normal pair, DNA was extracted from non-tumour tissue and microdissected tumour foci prepared from 10 μm paraffin-embedded sections as described previously (Shaw et al, 1996; Chappell et al, 1997).

**Table 1** Clinicopathological features of early breast cancers showing alterations at *IGF2R*

| Case | Type | Grade | Alteration |
|---|---|---|---|
| D2 | DCIS | High | LOH |
| D4 | DCIS | Low | LOH |
| D13 | DCIS | High | LOH |
| D14 | DCIS | High | LOH |
| 55 | IDC | II | MSI |

DCIS, ductal carcinoma in situ; IDC, infiltrating ductal carcinoma; LOH, loss of heterozygosity; MSI, microsatellite instability.

### PCR analysis at IGF2R

PCR reaction components were as follows: 45 mM Tris hydrochloric acid, pH 8.8; 11 mM ammonium sulphate; 4.5 mM magnesium chloride, 200 μM dTTP; dCTP; dGTP; 25 μM dATP (Pharmacia, UK); 0.2 μl [α-$^{35}$S]deoxyadenosine-5′-triphosphate (600 Ci mmol$^{-1}$, 10 mCi ml$^{-1}$ (ICN Pharmaceuticals, UK); 113 μg ml$^{-1}$ bovine serum albumin (Boehringher Mannheim); 6.7 mM β-mercaptoethanol; 4.4 μM EDTA, pH 8.0; 10 pmol of both the forward (GTA TCA TGA GAA CCT GAA GAG) and the reverse primer (TTG CCG GCT GGT GAA TTC AA) (Hol et al, 1992); 100 ng of DNA or 2 μl of microdissected DNA and 1 unit of *Taq* DNA polymerase (Gibco BRL, UK) in a total volume of 25 μl. Hot-start PCR was carried out using the following: 5 min denaturation at 94°C, followed by 30 cycles of 1 min denaturation at 94°C, 1 min annealing at 65°C, and 1 min extension at 72°C with a final extension of 7 min at 72°C on a DNA Thermal Cycler (Perkin Elmer Cetus, UK). Analysis of PCR products and interpretation of LOH were as described previously (Shaw et al, 1996, Chappell et al, 1997).

### RESULTS

We have analysed 62 'early' breast carcinomas comprising 40 invasive carcinomas and 22 cases of pure DCIS for LOH at *IGF2R*. Because of the complex heterogeneity of the disease and the presence of non-tumour cells, we carried out LOH analysis on DNA extracted from different foci that had been microdissected from within the same tumour tissue section. Cases that were homozygous at the *IGF2R* polymorphic repeat (Figure 1A) were considered uninformative. The frequency of heterozygosity varied between the two groups: 25 of the 'early' invasive cases (62.5%) and 18 of the 22 preinvasive cases of DCIS (82%) were informative. Of the total 43 cases (69%) that were informative five showed alterations at *IGF2R*. The clinicopathological features of these cases are summarized in Table 1.

The group of 'early' invasive carcinomas were predominantly well- or moderately differentiated cases (95% grade 1 or grade 2). None of these tumours showed any evidence of LOH at *IGF2R*, although one cases showed clear evidence of microsatellite instability (Figure 1B). In contrast, 4 of the 18 informative DCIS cases (22%) showed clear evidence of LOH. For example, Figure 1C shows DCIS cases 2 that exhibited loss of both the upper and lower allele in separate microdissected tumour ducts. Figure 1D shows DCIS case 4 with clear loss of the upper allele in all ducts examined. Three of the four cases of DCIS with LOH were high nuclear-grade cases, and one case was low grade (Table 1), suggesting an association between LOH at *IGF2R* and poor differentiation in the early stages of breast cancer development and progression.

© Cancer Research Campaign 1997

Figure 1 Representative examples of LOH at the *IGF2R* locus in 'early' breast cancers. N, normal tissue; T, tumour tissue. (**A**) Two non-informative invasive carcinoma patients; (**B**) informative early invasive carcinoma showing microsatellite instability; (**C** and **D**) DCIS cases 2 and 4, respectively, with LOH

### DISCUSSION

In this study we examined 'early' breast carcinomas for loss of heterozygosity at a polymorphic microsatellite locus within the 3′ untranslated region of the *IGF2R* gene on chromosome 6q26–27. DNA samples extracted from microdissected tumour foci prepared from 10 μm paraffin-embedded tumour tissue sections were examined separately. We found a difference in the frequency of LOH between the two groups of early lesions studied. None of the 40 early invasive carcinomas showed any evidence of allele loss, although one case showed microsatellite instability. This case has been reported previously and shows instability at nine out of ten other loci tested (Shaw et al, 1996). The group of 'early' invasive carcinomas were predominantly grade 1 or grade 2 cases (95%), suggesting no involvement or inactivation of *IGF2R* in well- to moderately differentiated tumours. In contrast, 4 out of 18 informative cases of DCIS showed clear evidence of LOH. Although the DCIS lesions represent a pre-invasive stage of breast cancer, 10 out of the 22 cases examined were high nuclear grade, which is recognized as a more aggressive form of the disease (Lagios, 1990). Three of the four DCIS tumours that showed LOH were from the ten high-grade cases, suggesting an association between inactivation of *IGF2R* and poorly differentiated in situ lesions. These data support the findings of Hankins et al (1996), who reported LOH with missense mutations in the remaining allele in two comedo-type (high-grade) DCIS cases. In combination, these data suggest that inactivation of *IGF2R* may occur only within certain more aggressive subgroups (poorly differentiated cases) of these 'early' breast cancers. Unfortunately, we were not able to undertake sequence analysis of the remaining allele in those cases that showed LOH because of the paucity of available DNA, prepared by microdissection, for analysis of the large *IGF2R* gene.

The *IGF2R* gene was first identified as a tumour-suppressor gene in hepatocellular tumours (De Souza et al, 1995*b*) and the presence of LOH at the *IGF2R* locus in adenomas suggests that inactivation may be an early event in liver carcinogenesis. The data reported in this paper, taken together with that of Hankins et al (1996), would support a similar early involvement of *IGF2R* inactivation in certain pathways in the development and progression of breast cancer. Additional evidence, suggesting a role for the *IGF2R* gene in mammary carcinogenesis, comes from two other key investigations. Jirtle et al (1993) first demonstrated that steady-state *IGF2R* mRNA levels in rat mammary tumours, regressing in response to d-limonene, increased twofold when compared with untreated tumours and that in unresponsive tumours expression of *IGF2R* was unaltered. More recently, Ellis et al (1996), have shown that the affinity of IGF2 for *IGF2R* inhibits IGF2 activity in MCF-7 breast cancer cells. Cellular proliferation, receptor tyrosine kinase-dependent signalling and extracellular IGF2 protein accumulation were all reduced specifically in the presence of IGF2R affinity. Therefore, by operating as an IGF2 anatagonist the *IGF2R* gene has tumour suppressor-like properties.

The lack of detection of LOH in the early invasive group of cases does not appear to result from technical problems in interpretation of LOH data (e.g. masking of any lost alleles by contaminating non-tumour material) as the same DNA samples prepared by microdissection from these carcinomas show clear LOH with three other microsatellite markers (ESR, D6S186, D6S193) that map to 6q25.1–q27 (Chappell et al, 1997). Moreover, the group of DCIS cases has previously been studied and also showed more frequent LOH (50%) with the three other markers. The frequent LOH detected within 6q25.1–27 could therefore indicate the critical inactivation of other unknown tumour-suppressor genes within this chromosomal interval (Chappell et al, 1997).

One factor that might interfere with the detection of LOH in this study is when polymerase amplification of dinucleotide repeats produces slippage bands below the true allele (Louis et al, 1992). Given that for a high proportion of the cases heterozygous for the *IGF2R* dinucleotide repeat, the two alleles differed by only 2 bp in length, any slippage bands would tend to mask loss of the smaller allele and hence reduce the true frequency of allelic loss. However, one of our DCIS cases that showed LOH (Figure 1C) had clearly lost both the upper and lower allele in different microdissected tumour ducts. Other mechanisms that do not involve LOH but also lead to inactivation of tumour-suppressor genes might also be critical for inactivation of *IGF2R* in breast carcinogenesis. For example, aberrant hypermethylation of 5′ CpG islands within proximal promoter regions has been implicated as a mechanism by which tumour-suppressor genes can be inactivated. This has been demonstrated for *E-cadherin* (Graff et al, 1995) and for the *VHL* and *p16* tumour-suppressor genes (Herman et al, 1994; Merlo et al, 1995). Therefore, it would be of interest to investigate the CpG island methylation status within the 5′ regulatory region of the *IGF2R* gene.

Our study has been concerned with breast cancers at an 'early' stage: small, node-negative invasive cases that have features associated with a good prognosis appear to show no evidence of LOH at *IGF2R*, whereas high-grade cases of DCIS although at a pre-invasive stage show evidence of LOH. These data provide good evidence that *IGF2R* acts as a tumour-suppressor gene in the development of some early breast cancers associated with a more aggressive disease type.

© Cancer Research Campaign 1997

### ACKNOWLEDGEMENTS

Tom Walsh is a PhD student supported by the Department of Pathology and the University of Leicester. This work was supported by funding from the Glenfield Hospital Research Committee.

### REFERENCES

Chappell SA, Walsh T, Walker RA and Shaw JA (1997) Loss of heterozygosity at chromosome 6q in preinvasive and early invasive breast carcinomas. *Br J Cancer* **75**: 1324–1329

Cullen KJ, Yee D, Sly WS, Pardue J, Hampton B, Lippman ME and Rosen N (1990) Insulin-like growth factor expression and function in human breast cancer. *Cancer Res* **50**: 48–53

De Leon DD, Bakker B, Wilson DM, Hitz RL and Rosenfeld RG (1988) Demonstration of insulin-like growth factor (IGF-I and -II) receptors and binding protein in human breast cancer cell lines. *Biochem Biophys Res Commun* **152**: 398–405

Dennis PA and Rifkin DB (1991) Cellular activation of latent transforming growth factor β requires binding to the cation-independent mannose 6-phosphate/insulin-like growth factor type II receptor. *Proc Natl Acad Sci USA* **88**: 580–584

De Souza AT, Hankins GR, Washington MK, Fine RL, Orton TC and Jirtle RL (1995*a*) Frequent loss of heterozygosity on 6q at the mannose 6-phosphate/insulin-like growth factor II locus in human hepatocellular tumours. *Oncogene* **10**: 1725–1729

De Souza AT, Hankins GR, Washington MK, Orton TC and Jirtle RL (1995*b*). M6P/IGF2R gene is mutated in human hepatocellular carcinomas with loss of heterozygosity. *Nature Genet* **11**: 447–449

Devilee P, van Vliet M, van Sloun P, Kuipers Dijkshoorn N, Hermans J, Pearson PL and Cornelisse CJ (1991) Allelotype of human breast carcinoma: a second major site for loss of heterozygosity is on chromosome 6q. *Oncogene* **6**: 1705–1711

Ellis MJC, Singer C, Hornby A, Rasmussen A and Cullen KJ (1994) Insulin-like growth factor mediated stromal-epithelial interactions in human breast cancer. *Breast Cancer Res Treat* **31**: 249–261

Ellis MJC, Leav BA, Yang Z, Rasmussen A, Pearce A, Zweibel JA, Lippman ME and Cullen KJ (1996) Affinity for the Insulin-Like Growth Factor II (IGF-II) Receptor inhibits autocrine IGF-II activity in MCF-7 breast cancer cells. *Mol Endocrinol* **10**: 286–297

Elston CW and Ellis IO (1991) Pathological prognostic factors in breast cancer. I. The value of histological grade in breast cancer: experience from a large study with long-term follow-up. *Histopathology* **19**: 403–410

Graff JR, Herman JG, Lapidus RG, Chopra H, Xu R, Jarrard DF, Isaacs WB, Pitha PM, Davidson NE and Baylin SB (1995) E-cadherin expression is silenced by DNA hypermethylation in human breast and prostate carcinomas. *Cancer Res* **55**: 5195–5199

Hankins GR, De Souza AT, Bentley RC, Patel MR, Marks JR, Iglehart JD and Jirtle RL (1996) M6P/IGF2 receptor: a candidate breast tumour suppressor gene. *Oncogene* **12**: 2003–2009

Hébert E, Herbelin C and Bougnoux P (1994) Analysis of the IGF-II receptor gene copy number in breast carcinoma. *Br J Cancer* **69**: 120–124

Herman JG, Latif F, Weng Y, Lerman MI, Zbar B, Liu S, Samid D, Duan DSR, Gnarra JR, Linehan WM and Baylin SB (1994) Silencing of the VHL tumour-suppressor gene by DNA methylation in renal carcinoma. *Proc Natl Acad Sci USA* **91**: 9700–9704

Hol FA, Geurds MP, Hamel BC and Mariman ECM (1992) Improving the polymorphism content of the 3′ UTR of the human IGF2R gene. *Hum Mol Genet* **1**: 347

Jirtle RL, Haag JD, Ariazi EA and Gould MN (1993) Increased mannose phosphate/insulin-like growth factor II receptor and transforming growth factor β1 levels during monoterpene-induced regression of mammary tumours. *Cancer Res* **53**: 3849–3852

Kornfeld S (1992) Structure and function of the mannose 6-phosphate/insulin like growth factor II receptor. *Annu Rev Biochem* **61**: 307–330

Lagios MD (1990) Duct carcinoma in situ: pathology and treatment. *Surg Clin N Am* **70**: 853–871

Laureys G, Barton DE, Ullrich A and Francke U (1988) Chromosomal mapping of the gene for the type II insulin-like growth factor receptor/cation-independent mannose 6-phosphate receptor in man and mouse. *Genomics* **3**: 224–229

Louis DN, von Deimling A and Seizinger BR (1992) A $(CA)_n$ dinucleotide repeat assay for evaluating loss of allelic heterozygosity in small and archival human brain tumour specimens. *Am J Pathol* **141**: 777–782

Menasce LP, Orphanous V, Santibanez-Koref M, Boyle JM and Harrison CJ (1994) Common region of deletion on the long arm of chromosome 6 in non-Hodgkin's lymphoma and acute lymphoblastic leukaemia. *Genes Chrom Cancer* **10**: 286–288

Merlo A, Gabrielson E, Mabry M, Vollmer R, Baylin SB and Sidransky D (1994) Homozygous deletion on chromosome 9p and loss of heterozygosity on 9q, 6p, and 6q in primary human small cell lung cancer. *Cancer Res* **54**: 2322–2326

Merlo A, Herman JG, Mao L, Lee DJ, Gabrielson E, Burger PC, Baylin SB and Sidransky D (1995) 5′ CpG island methylation is associated with transcriptional silencing of the tumour suppressor p16/CDKN2/MTS1 in human cancers. *Nature Med* **1**: 686–692

Millikin D, Meese E, Vogelstein B, Witkowski C and Trent J (1991) Loss of heterozygosity for loci on the long arm of chromosome 6 in human malignant melanoma. *Cancer Res* **51**: 5449–5453

Morita R, Saito S, Ishikawa J, Ogawa O, Yoshida O, Yamakawa K and Nakamra Y (1991) Common regions of deletion on chromosome 5q, 6q, and 10q in renal cell carcinoma. *Cancer Res* **51**: 5817–5820

National Coordinating Group for Breast Screening Pathology (1995) *Pathology Reporting in Breast Cancer Screening*, 2nd edn. NHSBSP: Sheffield, UK

Orphanos V, McGown G, Hey Y, Boyle JM and Santibanez-Koref M (1995) Proximal 6q, a region showing allele loss in primary breast cancer. *Br J Cancer* **71**: 290–293

Paik S (1992) Expression of IGF-I and IGF-II mRNA in breast tissue. *Breast Cancer Res Treat* **22**: 31–38

Rodabaugh KJ, Blanchard G, Welch WR, Bell DA, Berkowitz RS and Mok SC (1995) Detailed deletion mapping of chromosome 6q in borderline epithelial ovarian tumors. *Cancer Res* **55**: 2169–2172

Singer C, Rasmussen A, Smith HS, Lippman ME, Lynch HT and Cullen KJ (1995) Malignant breast epithelium selects for insulin-like growth factor II expression in breast stroma: evidence of paracrine function. *Cancer Res* **55**: 2448–2454

Shaw JA, Walsh T, Chappell SA, Carey N, Johnson K and Walker RA (1996) Microsatellite instability in early sporadic breast cancer. *Br J Cancer* **73**: 1393–1397

Zhoa Y, Escot C, Maudelonde T, Puech C, Rouanet P and Rochefort H (1993) Correlation between mannose-6-phosphate/IGFII receptor and cathepsin D RNA levels by *in situ* hybridization in benign and malignant mammary tumours. *Cancer Res* **53**: 2901–2905

© Cancer Research Campaign 1997